# Tibial access for supra-inguinal embolization in extremely obese patients

**DOI:** 10.1186/s42155-020-00105-6

**Published:** 2020-03-09

**Authors:** Jason C. Smith, Alex L. Cho, Scott T. Fujimoto

**Affiliations:** Department of Radiology, Loma Linda University, 11234 Anderson Street, Loma Linda, California, 92354 USA

**Keywords:** Access, Tibial, Embolization, Trauma, Obesity

## Abstract

Extreme obesity is a risk factor for hemorrhagic complications of femoral access (FA). Femoral lines, hematomas, pelvic binders and coagulopathy in the trauma scenario may also add difficulty and/or risk to FA. Radial access (RA) for routine peripheral endovascular procedures has been popularized owing to decreased hemorrhagic complications, increased patient satisfaction, and decreased operator radiation dose. However, though uncommon, cerebrovascular complications from RA approach are a known risk. Relatively recently, tibial access (TA) has been used for lower extremity peripheral vascular disease interventions. The advantages of TA mirror that of RA, with few and mostly minor complications, and the risk of iatrogenic cerebral embolization is nil. We report the feasibility of TA for supra-inguinal embolization in two extremely obese patients {body mass index > 40 kg/m^2^} following motor vehicle accidents. Commercially available base and microcatheters were used to perform embolization of the affected lower abdominal or pelvic arteries in standard fashion via a novel trans-tibial artery approach.

## Background

Extreme obesity is a risk factor for hemorrhagic complications of femoral access (FA) (Hibbert et al. 2012). Femoral lines, hematomas, pelvic binders and coagulopathy in the trauma scenario may also add difficulty and/or risk to FA. In recent years, radial access (RA) for routine peripheral endovascular procedures has been popularized owing to decreased hemorrhagic complications (Posham et al. 2016) as well as improved patient satisfaction and decreased operator radiation dose (Yamada et al. 2018). However, not all investigators have found RA superior to traditional FA (Hung et al. 2019), and though uncommon, cerebrovascular complications from RA approach are a known risk. Stroke has been reported from peripheral angiography via RA (Al-Hakim et al. 2017), and compared to FA, patients undergoing cardiac angiography procedures from RA have been shown to have 2.1 times increased risk of silent stroke (Göksülük et al. 2018). Relatively recently, tibial access (TA) has been used for lower extremity peripheral vascular disease interventions (Montero-Baker et al. 2008; Walker et al. 2016; Sanghvi et al. 2018; Kwan et al. 2015). We report the feasibility of TA for supra-inguinal embolization in two extremely obese patients {body mass index (BMI) > 40 kg/m^2^}.

### Tibial access for embolization of Suprainguinal hemorrhage

Two patients with pelvic or abdominal wall hemorrhage following motor vehicle accidents were treated at our institution with trans-catheter embolization via TA. Patencies of tibio-pedal arteries {anterior tibial (ATA), posterior tibial (PTA), dorsalis pedis (DPA)} were documented pre-procedurally with palpation and/or ultrasound. With ultrasound guidance and micropuncture technique, a 5 French Glidesheath Slender (Terumo, USA) was placed, hand injection of contrast via the sheath was performed, and 2000–3000 IU of heparin and 200 μg of nitroglycerin were instilled through the sheath as previously reported for RA (Yamada et al. 2018). An additional 1500 IU of heparin was administered 2 h later to our first described patient immediately before procedure’s termination. Access site hemostasis was achieved with a large TR band radial artery compression device (Terumo, USA) reinforced with cloth tape. This study was performed in compliance with our Institutional Review Board, and patient consent for publication of their cases was obtained.

### Case 1

**Fig. 1:**
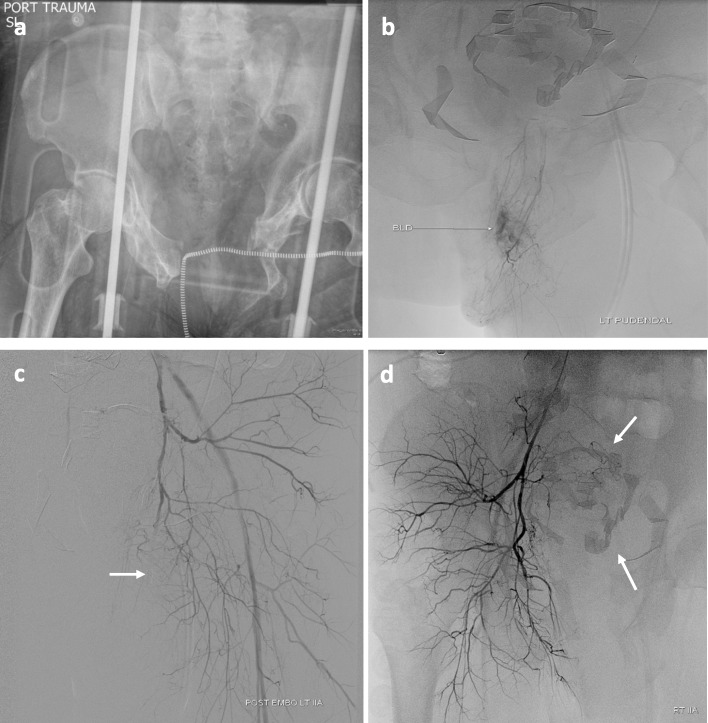
Patient 1 Initial angioembolization procedure via a trans-radial approach: AP Pelvis radiograph showing open book pelvic fractures (**a**). Superselective left pudendal artery angiogram (**b**). Follow-up left internal iliac angiogram showing coil occluded (arrow) left pudendal artery, reflux into the external iliac artery, and no other signs of hemorrhage in the left hemi-pelvis (**c**). Negative right internal iliac angiogram also showing reflux into the external iliac artery. Note lap sponges in the mid pelvis (arrrows) (**d**)

A 51-year-old male with a BMI of 42.3 kg/m^2^ suffered a motorcycle accident. On admission, his blood pressure was 92/51 mmHg, his heart rate was 134 beats/min, and his hemoglobin (Hgb) declined from 14.2 to 11.1 g/dL despite 8 units of blood products. Radiography demonstrated open book pelvic fractures (Fig. 1a), and focused assessment with sonography for trauma (FAST) examination demonstrated free intraperitoneal fluid consistent with hemorrhage. He was transported emergently to the operating room for exploratory laparotomy where bladder rupture was identified and pelvic packing was performed, but due to continued uncontained pelvic hemorrhage he was placed in a pelvic binder and brought to Interventional Radiology (IR). Via left RA, pelvic angiography was performed, and an actively extravasating left pudendal artery (Fig. 1b) was coil embolized; no other bleeding source was identified (Fig. 1c, d). Following sheath removal, patent hemostasis was achieved with a TR band. CT of the head, neck and torso was performed following the angioembolization. CT demonstrated intra- and extra-peritoneal contrast extravasation thought related to bladder injury, but did not show definitive active vascular extravasation.

However, overnight he had continued hemodynamic instability and blood drainage through his negative pressure wound therapy device on his anterior abdominal wound, necessitating blood product replacement. Therefore, he was returned to IR for empiric embolization of the bilateral internal iliac arteries due to high level of suspicion for active pelvic bleeding prior to planned orthopedic pelvic stabilization surgery later that day. Physical examination revealed an occluded left radial artery with patent ulnar artery and good hand perfusion.

**Fig. 2:**
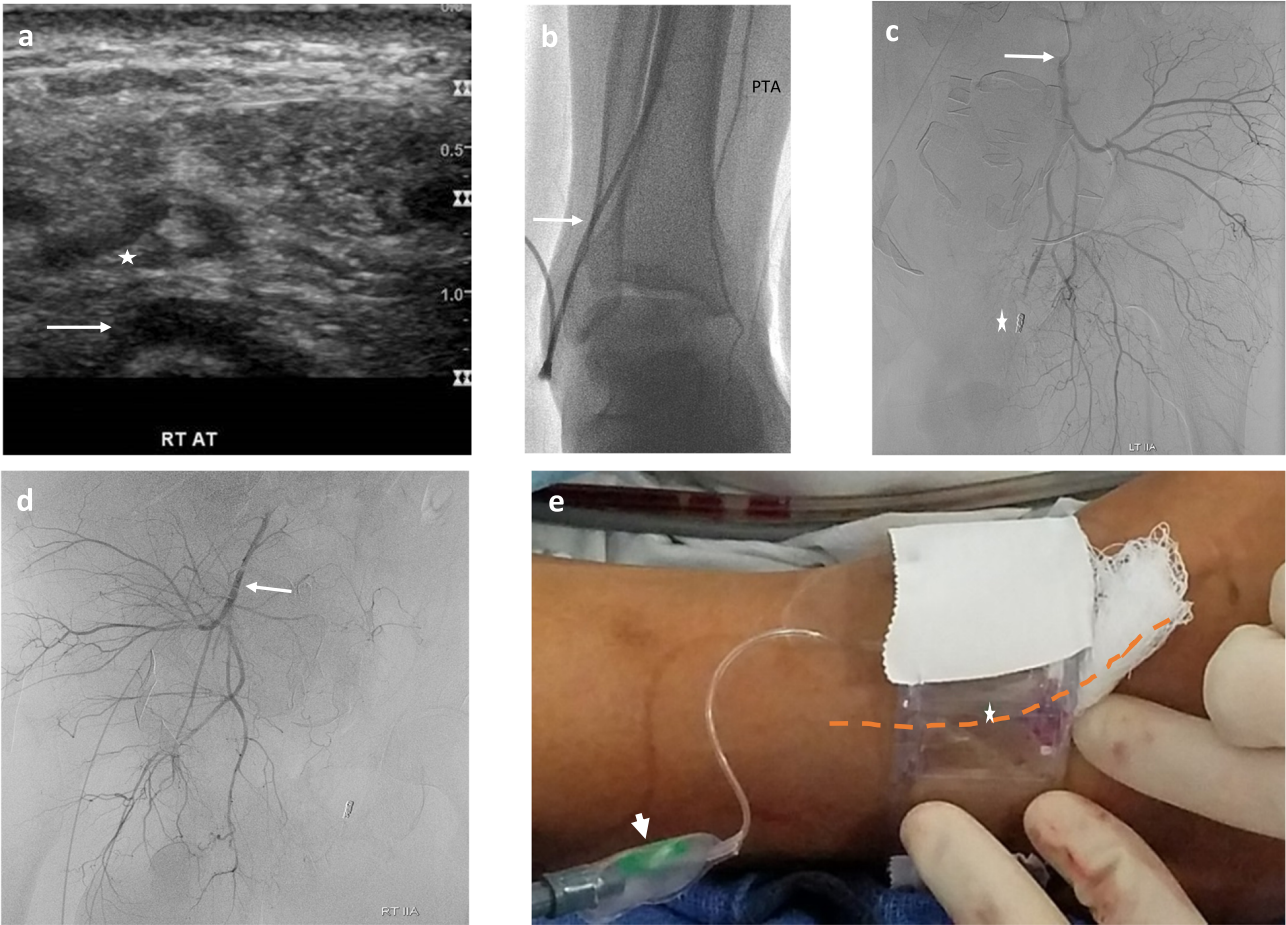
Patient 1 Subsequent angioembolization procedure via a trans-tibial approach: Ultrasound guided access into the distal right ATA (*) over the distal tibial cortex (arrow). Hand injection through vascular sheath following ATA access (arrow) showing reflux into the PTA (**b**). Left internal iliac angiogram via base 0.035 catheter (arrow) demonstrates occluded left pudendal artery (*), and no signs of hemorrhage in the left hemi-pelvis (**c**). Right internal iliac angiogram via base 0.035 catheter (arrow) was negative for hemorrhage (**d**). Hemostasis of the ATA (− − - indicates underlying arterial course) was achieved with a large TR band (* indicates balloon; arrowhead points to inflation port) buttressed by a single piece of cloth tape (**e**)

Via the 2.5 mm diameter distal right ATA (Fig. 2a, b), a steam-shaped 5 French 130 cm Mariner catheter (Angiodynamics, USA) was advanced into the contralateral left internal iliac artery where a negative angiogram was obtained (Fig. 2c). A Renegade STC microcatheter (Boston Scientific, USA) was advanced coaxially into its anterior division, and embolization was performed with Gelfoam slurry (Pfizer Pharmacia & Upjohn, USA) capped by platinum coils. A Waltman loop was formed with the base catheter and its tip repositioned into the right internal iliac artery, where a negative angiogram was obtained (Fig. 2d). The microcatheter was advanced into its anterior division, and Gelfoam slurry embolization was performed to stasis. Following sheath removal, patent hemostasis was achieved with a TR band (Fig. 2e). Post-procedurally, he stabilized. One year follow-up physical examination revealed mildly decreased sensation over the dorsal radial aspect of his left hand and a strong right ATA pulse.

### Case 2

**Fig. 3:**
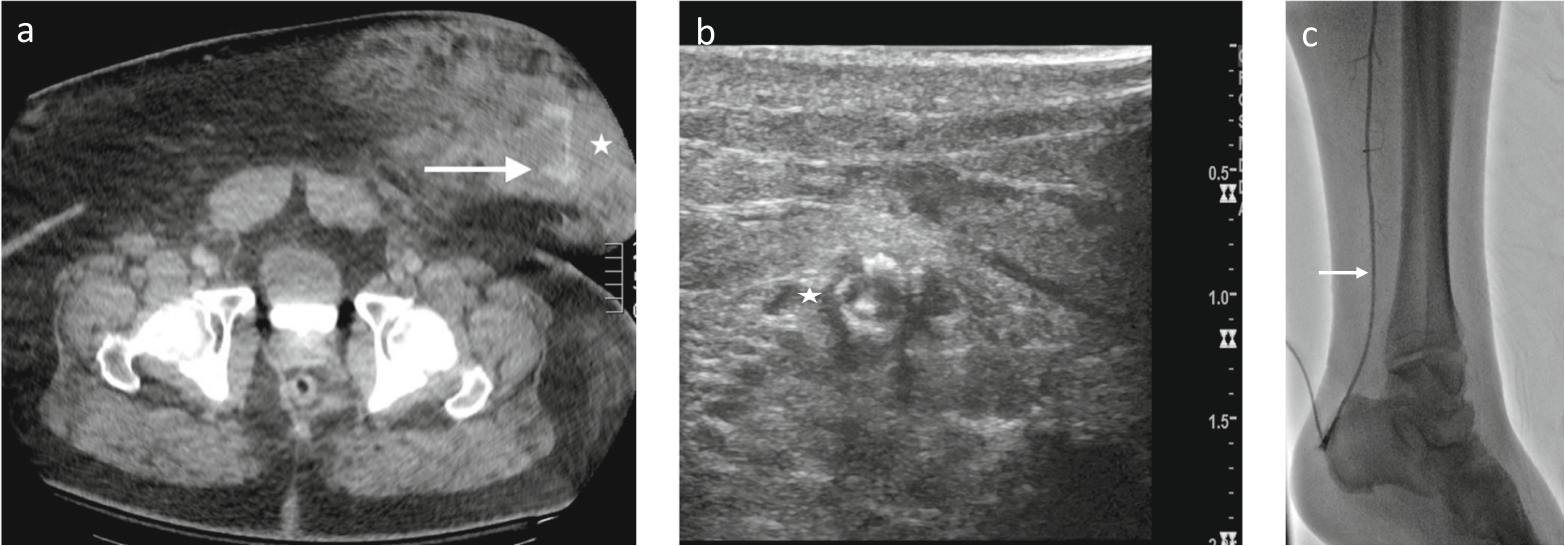
Patient 2 Seat-belt injury and ipsilateral tibial access: Axial contrast enhanced CT following seat-belt injury demonstrating contrast extravasation (arrow) in the left anterior abdominal hematoma (*) (**a**). Ultrasound guided access into the distal PTA (*) (**b**). Hand injection through vascular sheath following PTA access (**c**)

A 48-year-old male driver with a BMI of 50.6 kg/m^2^ suffered an automobile accident while wearing a seatbelt. On admission, his blood pressure was 58/51 mmHg leading to vasopressor support, and his Hgb dropped from 14.3 to 11.4 g/dL. CT revealed active hemorrhage in the lower left anterolateral abdominal wall (Fig. 3a), for which a pelvic binder was placed.

**Fig. 4:**
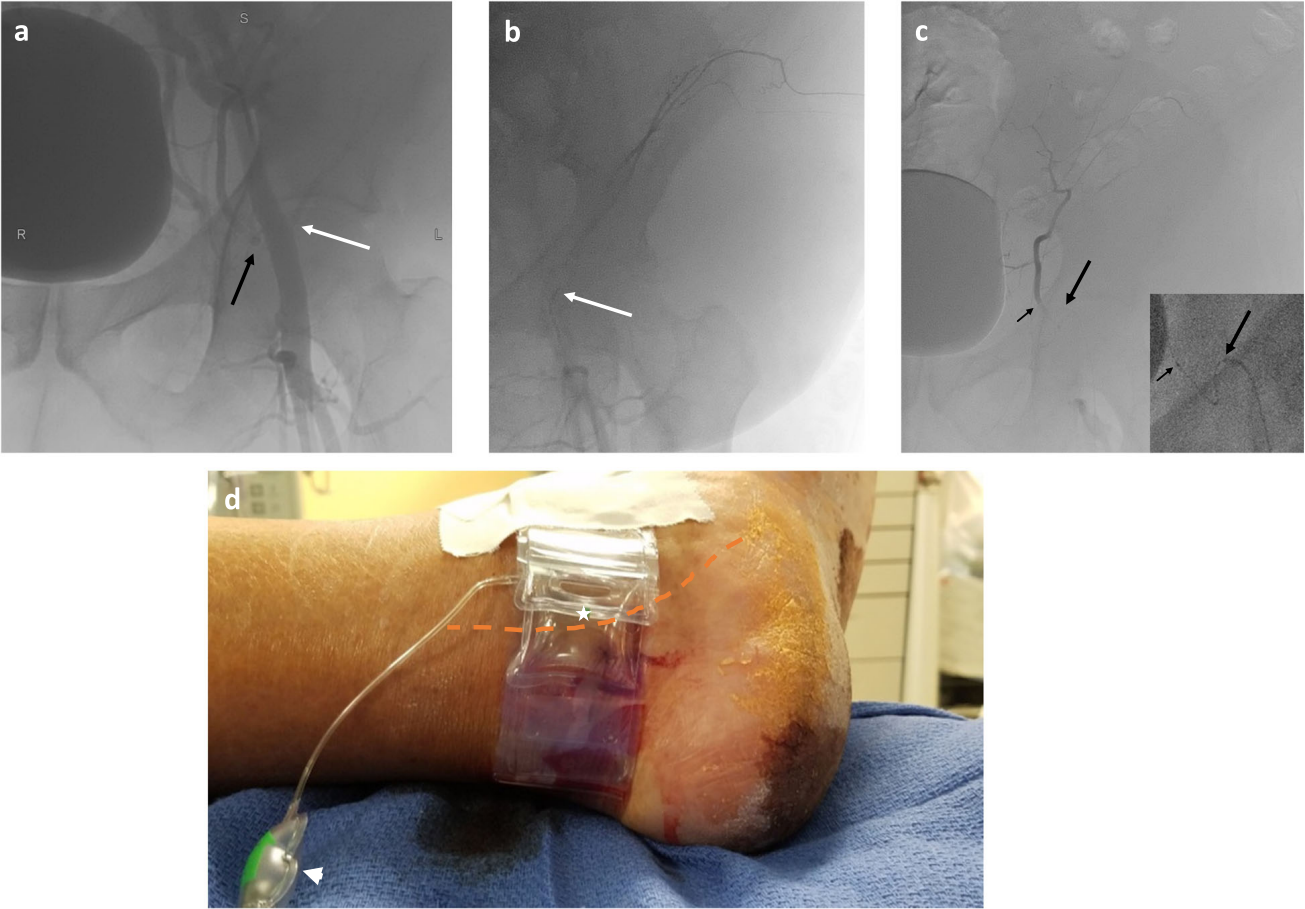
Patient 2 Angioembolization: Hand injection left external iliac angiogram to delineate the origins of the ipsilateral abdominal wall arteries (**a**). Deep circumflex iliac angiogram without active extravasation, which was subsequently embolized to stasis with Gelfoam slurry (**b**). Inferior epigastric angiogram (inset shows fluoroscopic last image hold; long arrow points to base catheter, short arrow points to microcatheter) without active extravasation; this vessel was also embolized to stasis with polyvinyl alcohol particles (**c**). Hemostasis of the PTA (− − - indicates underlying course) was achieved with a large TR band (* indicates balloon; arrowhead points to inflation port) buttressed by a single piece of cloth tape (**d**)

Via the 3.4 mm diameter left PTA (Fig. 3b, c), a 5 French 100 cm KMP (Cook Medical, USA) was advanced into the ipsilateral external iliac artery and hand injection angiography performed (Fig. 4 a). The KMP catheter was then advanced into the deep circumflex iliac artery (DCIA) and negative angiogram was obtained (Fig. 4b). Superselective access was achieved more distally within this vessel with a Renegade STC microcatheter, and Gelfoam slurry embolization was performed. Next, the inferior epigastric artery (IEA) was catheterized with a 5 French 100 cm Judkins right 4 (Cordis, USA) catheter. Superselective access was achieved more distally within this vessel with the micro-catheter, through which a negative angiogram was obtained (Fig. 4c); the IEA was then embolized with 355–500 um polyvinyl alcohol particles (Boston Scientific, USA). Following sheath removal, patent hemostasis was achieved with a TR band (Fig. 4d).

Post-procedurally, he stabilized. Ultrasound the following day revealed a patent left PTA, and he was subsequently transferred to a rehabilitation hospital. Patient stated on phone follow-up 11 months later that he was ambulating without difficulty.

## Discussion of technique

The advantages of TA mirror that of RA, with few and mostly minor complications, and the risk of iatrogenic cerebral embolization is nil (Montero-Baker et al. 2008; Walker et al. 2016; Sanghvi et al. 2018; Kwan et al. 2015). Patency of tibial arteries should be confirmed pre-procedurally to minimize rare ischemic complications to the foot should occlusion from dissection or thrombosis of the accessed artery occur. The calibers of tibial arteries are generally as large as the wrist arteries. The distal ATA may be preferred compared to the PTA, owing to its intimate course over the distal tibia. Steam-shaping a 130 cm base catheter tip to navigate over the aortic bifurcation was successful. A proprietary pre-curved, “cobra” shaped catheter of approximately 130 cm length would be helpful both for crossing the aortic bifurcation and for forming a Waltman loop for accessing the ipsilateral internal iliac artery. Standard, proprietary base and microcatheters are commercially available in adequate lengths to reach the contralateral internal iliac artery in average height adult patients. Patent hemostasis with application of a large TR compression band is easily assessed after TA. Finally, though the patients described in this report fortunately did not suffer neurological consequence, currently we do not administer prophylactic anti-coagulation to minimize thrombotic access complications in our acute blunt trauma patients who may have potential closed head injury with its attendant risk of inciting or exacerbating intra-cranial hemorrhage.

## Conclusion

We report the feasibility of a trans-tibial approach as an alternative arterial access in two extremely obese patients with pelvic and lower abdominal wall hemorrhage. Diagnostic trauma CT imaging routinely obtained is essential for demonstrating the location of hemorrhaging sites as well as for evaluating the iliofemoral artery conduits to be navigated. A limitation of this report includes the patients’ relatively young age without tortuous or atherosclerotic arteries, through which torquability may be expected to be compromised. Additionally, angiographic studies were limited to the pelvis and lower abdominal wall; had angio-intervention been necessary from unexpected intra-abdominal hemorrhage unidentified on CT, conversion to FA or RA would likely have been necessary due in part to catheter length considerations. Further experience would be necessary to demonstrate the feasibility of TA as an intriguing alternative access for other supra-inguinal, non-peripheral limb ischemia interventions.

## Data Availability

All data generated or analyzed during this study are included in this published article.

## Abbreviations

ATA: Anterior tibial artery
BMI: Body mass index
DPA: Dorsalis pedis artery
FA: Femoral access
IR: Interventional Radiology
PTA: Posterior tibial artery
RA: Radial access
TA: Tibial access
